# Exploring Reddit Discourse and Information Needs Surrounding Extreme Heat: Topic, Sentiment, and Engagement Analysis

**DOI:** 10.2196/82426

**Published:** 2026-02-25

**Authors:** Melissa MacKay, Soroush Zamani Alavijeh, Sydney Gosselin, Fattane Zarrinkalam, Jennifer E McWhirter

**Affiliations:** 1 Department of Population Medicine, University of Guelph, 50 Stone Rd E, Guelph, ON, N1G2W1, Canada, 1 5198244120; 2 Department of Computing and Software, McMaster University, Hamilton, ON, Canada; 3 College of Engineering, University of Guelph, Guelph, ON, Canada

**Keywords:** social media, Reddit, climate change, extreme heat, information ecosystem, health communication

## Abstract

**Background:**

As Canada’s climate changes, extreme heat events have become more frequent, a trend that is expected to continue. Extreme heat can lead to several negative health outcomes, which disproportionately impact vulnerable populations. Evidence-based, equitable interventions are needed to inform and protect the public from the health effects. Effective communication can aid this effort to improve health outcomes by emphasizing the connection between health risks and climate change and empowering people to act. Machine learning has applications in understanding current attitudes, beliefs, experiences, and behaviors within the target audience for public health messaging. Machine learning analyses of social media data have elucidated user perceptions of heat events in the literature; however, research is limited with respect to social media user perceptions, beliefs, and behaviors related to extreme heat, particularly in the Canadian context. Analyzing Canadian social media discourse related to extreme heat will help to address this research gap and inform future research and communications to reduce the risks of extreme heat.

**Objective:**

The purpose of this research is to better understand Canadian discourse and emotions related to extreme heat by examining social media (Reddit). Our objectives include (1) identifying common discussion topics, concerns, and questions related to extreme heat among Canadian Reddit users; (2) analyzing sentiment and emotional responses to extreme heat discussions; and (3) investigating the relationship between topics, sentiment, and engagement for posts.

**Methods:**

We collected data using the Reddit application programming interface (API), retrieving posts from 30 Canada-specific subreddits between February 12, 2023, and February 11, 2024, based on a predefined set of heat- and climate-related keywords. Posts and comments were structured as hierarchical tree models, with text consolidated into documents for analysis. Topic modeling, sentiment analysis, and emotion analysis were conducted; engagement was assessed using net upvote scores to gauge community approval.

**Results:**

The analysis of 607 Reddit posts from 15,366 users revealed that discussions about extreme heat were most frequently centered around the keyword “heat,” which appeared in 82.5% (n=501) of the posts and 81.1% (n=25,253) of the comments. Topic analysis identified key themes related to heating and cooling costs, weather records, air conditioning, and health impacts, while sentiment and emotion analyses showed varying levels of positivity and negativity across subreddits.

**Conclusions:**

Our findings present an initial snapshot into Canadian perspectives and information needs about extreme heat in Canada. In our sample, discussions on Reddit about extreme heat in Canada are dominated by concerns over heating and cooling costs, weather patterns, and personal adaptation strategies, reflecting both practical and policy-related challenges. Additionally, sentiment and emotion analyses suggest significant regional differences in public perception, which may be useful for informing health and risk messaging initiatives to better protect Canadians from the adverse health effects of climate change.

## Introduction

### Background

Climate change due to human activity has been driving shifts in temperatures and weather patterns [1]. Climate change has many consequences, including fires, flooding, droughts and water scarcity, and intense storms [1]. Because of these impacts, climate change affects health in diverse ways, and some individuals are more vulnerable to these health risks than others [1]. The extent of the impact on individuals is influenced by their exposure to hazards related to climate change, the degree of impact, and their capacity to cope with the climate hazard [2]. Infants, children, pregnant women, older adults, and persons with physical and mental impairments are more vulnerable to climate-related hazards [2]. Additionally, various social determinants of health, including socioeconomic status, race or racism, health care access, education, and the built environment, interact with climate change to produce patterns of disadvantage that lead to additional vulnerabilities [2].

In Canada, climate change is resulting in extreme weather events, including extreme heat, which has significant direct impacts on human health and the environment [3]. Direct health risks from extreme heat include the exacerbation of existing health conditions, heat exhaustion, heat stroke, and death [4]. Extreme heat can also intersect with the indirect health risks of a changing climate, which are related to the social determinants of health and can impact our food system, culture, housing, infectious disease distribution, water quality and availability, and the economic status of individuals and communities [5]. Indeed, extreme heat is one of the most deadly forms of extreme weather affecting Canadians and is a growing public health risk in Canada [6,7].

Effective communication about climate change–related health risks, including extreme heat, can improve individual and population-level health outcomes [8]. Evidence-based strategies for climate change communication emphasize highlighting health impacts, relying on trusted sources, minimizing barriers to adaptive behaviors, and leveraging social media to disseminate accurate information [8]. To support effective public health messaging, it is essential to understand public risk perceptions as well as their information needs and preferences regarding climate change [8].

Social media provides an important communication channel through which public health can share information and people can share their experiences and opinions [9]. Public health can potentially reach a larger audience, raise awareness and share information about climate-related hazards and issues, and mobilize action. It can also foster public discussion and positively impact attitudes toward climate policy [9,10]. Social media also presents challenges, including the spread of misinformation or disinformation, the creation of echo chambers, and the bias toward certain actors or ideologies because of algorithmic curation [10].

Social media provides a platform for public health professionals to not only reach wider audiences but also to monitor information, assess public awareness, and engage in ongoing discussions. Platforms such as Reddit, which has over 15.55 million users in Canada [11], offer valuable insights into public perceptions and discourse on health-related topics. Canadian users actively participate across subreddits, including r/canada, facilitating country-specific discussions. The large Canadian user base and the diversity of subreddits and topics discussed provide an important social media platform to assess extreme heat and climate-related discussions and trends to understand perceptions, sentiment, and information needs and wants. An analysis of 16 years of Reddit discussion suggests that trends in Reddit discussions are reflective of broader trends across Internet-based channels [12], further supporting the evaluation of Reddit to better understand discourse on climate change.

Although limited research has been conducted to examine climate change discourse on Reddit [13,14], no such research has been conducted with a focus on Canada. This study extends prior research on climate change discourse by offering a Canadian case study and demonstrating the potential of computational methods for examining emerging public concerns about extreme heat. To guide our study, we draw on principles from risk communication theory and the concept of the information ecosystem. Risk communication theory emphasizes the role of trusted channels, perceived risk, and emotional responses in shaping adaptive behaviors during climate hazards [8]. The information ecosystem perspective recognizes social media platforms, such as Reddit, as sites where diverse actors contribute to meaning-making, often shaping how risk information is sought, shared, and acted upon [10]. These perspectives position Reddit not only as a source of descriptive discourse but also as a meaningful part of how Canadians understand, interpret, and respond to extreme heat.

### Study Goal and Objectives

The goal of this research is to understand social media (Reddit) discourse and emotions related to extreme heat in Canada. Specific subobjectives of this research are as follows: (1) to identify common discussion topics, questions, and concerns related to extreme heat among Canadian Reddit users; (2) to analyze sentiment and emotional responses to extreme heat discussions; and (3) to investigate the relationship between topics, sentiment, and engagement for posts.

## Methods

This study was designed as a descriptive computational analysis, intended to provide an initial overview of Reddit discourse on extreme heat in Canada. As such, the results present descriptive outputs of topic, sentiment, emotion, and engagement analyses without qualitative interpretation.

### Ethical Considerations

Research ethics was not required as this study analyzed publicly available data only as per the University of Guelph’s Research Ethics Board [15]. Under Canada’s Tri-Council Policy Statement: Ethical Conduct for Research Involving Humans, Research Ethics Board review is not required for research that relies exclusively on publicly accessible information where individuals have no reasonable expectation of privacy; as our study analyzes only public Reddit posts without interaction or private identifiers, ethics review is not required [16]. No personally identifiable information or direct identifiers (eg, usernames, links, or IDs) were collected or analyzed as part of this study. No other personally identifiable information was collected or stored, such as user profile information or IP addresses.

### Data Source

To collect data for our analysis, we utilized the Reddit API [17] to search for posts within a selection of Canadian subreddits. These searches targeted posts that contained at least one of our designated keywords in their titles. Specifically, we examined 30 subreddits focused on national interests (eg, r/canada), provincial or territorial interests (eg, r/ontario, r/britishcolumbia), and local interests within provincial or territorial capital cities and most populous cities (if different; eg, r/vancouver, r/halifax, r/saskatoon). The Reddit posts included were made between February 12, 2023, and February 11, 2024. The data collected and analyzed were publicly available Reddit posts; however, our analyses did not use any personally identifiable information or direct identifiers (eg, usernames, links, or IDs).

### Data Acquisition

This research sought to understand prevalent topics, sentiment, and engagement information within Reddit posts and comments related to extreme heat events in Canada. Reddit users post content within designated communities, known as subreddits. When a user posts content in a subreddit, the other members of the subreddit can “upvote” (ie, indicate a favorable reaction), “downvote,” (ie, indicate an unfavorable reaction), and comment on the post. Subreddits can be focused on any topic or interest, including national, provincial or territorial, or local news and issues.

The research team collected posts and comments from a predetermined list of Canada-specific subreddits using Reddit’s API [17]. We used the Reddit API to search the selected subreddits for the following heat- and climate-related search terms: “heat,” “extreme heat,” “hot weather,” “hot summer,” “hottest day,” “heatwave,” “heat dome,” “hot day,” “hottest year,” “climate change AND heat,” and “BC heat wave.” These terms were determined after searching the subreddits for the keywords used to discuss extreme heat.

To ensure that our keyword set and subreddit sample appropriately captured discussion relevant to extreme heat in Canada, we conducted preliminary pilot searches. We manually browsed Canadian subreddits to identify the actual terms users used when discussing extreme heat, which helped refine our candidate keywords (eg, “heat dome,” “hottest day”) beyond generic terms, such as “hot weather.” For subreddit selection, we began with lists of national, provincial or territorial, and major city subreddits, then excluded those with very low activity (few heat-related posts during pilot months) or that were clearly irrelevant (eg, subreddits focused exclusively on entertainment or sports). This process ensured that the sample remained both manageable and relevant. Similar approaches are used in related social media studies, such as that by Janaswamy and Blackburn [18], who identified climate change–relevant subreddits by starting with seed communities and empirically validating activity levels, and that by Fariello and Jemielniak [12], who validated keyword sets through historical analyses of evolving climate-related terminology.

### Text Analytics

The textual data from Reddit posts and comments follow a hierarchical tree structure. Each discussion thread on Reddit begins with a main post, which forms the root node of the tree. Subsequent comments within the thread serve as child nodes. Specifically, a comment that directly responds to the main post is attached as a child to the root node. The comments that are replies to other comments are linked as child nodes to their respective parent comments. This recursive linkage continues, mapping the entire discussion thread into a tree structure where the path from the root to any node represents the flow of conversation from the initial post to a particular comment. Finally, the comments that have no replies are leaves of the tree with no children.

After constructing the tree structures to represent individual Reddit threads, we performed topic analysis on these threads. To facilitate effective topic detection by the language model, it is essential to create more cohesive documents that encapsulate the main context and key information within the discussions. This was achieved by traversing the tree in a level-order fashion. During this traversal, the text of each visited node (comment) was concatenated to form a continuous document. This process continued until the document reached a predefined size suitable for the input constraints of the language model. In this way, the comments that are replies to a specific comment were sequentially concatenated, preserving the flow of discussion within the document. The document construction was terminated either when it reached its maximum allowable size or when there were no further comments to process. If a cutoff occurred due to reaching the maximum size, a new document was initiated following the same methodology, continuing until all comments in the thread were processed. If a single comment or a single post was longer than the maximum size, it was constructed as one document by itself. This process began with the main Reddit post itself, considering it as the parent node from which all subsequent comments in the thread were derived and processed.

To mitigate the sparsity challenges of short comments and to preserve conversational flow, we concatenated comments and replies into larger “documents.” Similar strategies have been used in prior research to improve topic modeling and clustering of fragmented user-generated text. For example, Llewellyn et al [19] demonstrated that combining online newspaper comments with their replies produced more coherent topics when applying Latent Dirichlet Allocation . Likewise, Sun et al [20] developed a conversational structure-aware topic model, which leverages reply structures in threaded discussions to yield more interpretable topics. These approaches, like ours, show that aggregating related comments along conversational paths can enhance coherence while retaining contextual meaning that might be lost if each comment were treated entirely independently.

While traversing the 607 tree structures, we excluded comments generated by bots that were deleted or written in languages other than English. This filtering process removed 22,069 comments from the initial pool of 31,118, leaving 9049 valid comments along with the original 607 posts. These were consolidated into 1352 documents for analysis. On average, each document contained 270.56 words, aligning well with our language model’s input requirements, which accommodate a maximum token size of 384.

### Topic Analysis

For topic modeling, we used BERTopic (bidirectional encoder representations topic modeling), a neural topic modeling technique, to conduct topic analysis on the documents extracted from Reddit, which were assembled using the document consolidation method outlined in the text analytics section. BERTopic leverages class-based term frequency-inverse document frequency for improved topic representation [21]. BERTopic combines transformer-based embeddings with hierarchical clustering, enabling coherent topic extraction even from short or noisy texts, making it well-suited for our dataset. To assess coherence, we manually inspected representative documents for each identified topic to verify that the extracted clusters reflected consistent underlying themes.

### Sentiment Analysis

Sentiment analysis is used to ascertain the prevailing sentiment within textual data, specifically determining whether the sentiment of a given text is positive or negative, and assigning a score to quantify the intensity of the identified sentiment direction. We utilized the consolidated documents, assembled using the method described above, as input for the *distilbert-base-uncased-finetuned-sst-2-english* model [22,23]. This model was specifically designed for text classification tasks and was used to assess the sentiment direction and sentiment score for each document [24]. To provide a check on model performance, we also manually inspected a random sample of 50 documents classified as positive and 50 documents classified as negative. This validation suggested that the sentiment assignments generally aligned with the intended tone of the posts.

### Emotion Analysis

Emotion analysis is conducted on textual data to detect and identify various emotional elements within the text. For emotion analysis, we used roberta-base-go_emotions, an off-the-shelf model fine-tuned on the GoEmotions dataset [25]. RoBERTa’s optimized pretraining enhances contextual understanding, while fine-tuning on GoEmotions enables precise detection of fine-grained emotions [26,27]. GoEmotions dataset includes 12 positive, 11 negative, 4 ambiguous, and 1 neutral emotion, making it suitable for conversation and differentiating between subtle emotion expression [28]. Using this fine-tuned language model on the consolidated documents previously described, we discerned the level of negative (eg, anger, sadness, or fear) and positive (eg, admiration or optimism) emotions, as well as approval and disapproval emotions within the extracted Reddit documents.

### Engagement Analysis

Reddit users often express their agreement or disagreement with a given post or comment through upvotes and downvotes, respectively. The net upvote score of a Reddit post or comment serves as an indicator of engagement and is determined by subtracting the number of downvotes from the number of upvotes [29]. A post or comment with a high net upvote score is generally considered to be approved by the subreddit community, whereas one with a low net upvote score is viewed as disapproved [29].

## Results

### Data Collection

Based on the search terms and included subreddits, 607 Reddit posts were collected from over 15,000 users. On average, there were approximately 20 posts per subreddit and 52 comments per post. Table 1 provides the total number of posts, users, comments, and distribution of posts across the subreddits.

**Table 1.** Total posts, unique users, comments, comments per post, subreddits, and posts per subreddit.

Characteristic	Value
Reddit posts	607
Unique users	15,366
Comment	31,118
Comments per post, SD^a^	81.9
Comments per post, mean^a^	52.8
Comments per post, median (IQR)^a^	24.00 (9-58)
Subreddit	30
Posts per subreddit, SD	17.0
Posts per subreddit, mean	20.2
Posts per subreddit, median (IQR)	18.5 (6-29.5)

a Based on posts with at least 1 comment.

Across the 11 keywords used to identify posts in the included subreddits, “heat” was the most common (501 posts, 25,253 comments), followed by “extreme heat” (28 posts, 1320 comments) and “hot weather” (19 posts, 1561 comments; Table 2).

**Table 2.** Post and comment distribution across keywords.

Keyword	Post, n (%)	Comment, n (%)
heat	501 (82.5)	25,253 (81.1)
extreme heat	28 (4.6)	1,320 (4.2)
hot weather	19 (3.1)	1,561 (5)
hot summer	17 (2.8)	622 (2)
hottest day	11 (1.8)	344 (1.1)
heatwave	8 (1.3)	348 (1.1)
heat dome	7 (1.2)	868 (2.8)
hot day	6 (1)	225 (0.7)
hottest year	5 (0.8)	363 (1.1)
climate change AND heat	3 (0.5)	212 (0.7)
BC heat wave	2 (0.3)	2 (<0.01%)

Across the included subreddits, r/canada had the most posts and comments (62 and 6010, respectively), followed by r/halifax (53 posts, 1772 comments), and r/britishcolumbia (47 posts; 2927 comments). Table 3 shows the distribution of the posts and comments across all included subreddits.

**Table 3.** Post and comment distribution across subreddits.

Subreddit^a^	Post, n (%)	Comment, n (%)
r/canada	62 (10.2)	6010 (19.3)
r/halifax	53 (8.7)	1772 (5.7)
r/britishcolumbia	47 (7.7)	2927 (9.4)
r/vancouver	45 (7.4)	2475 (8.0)
r/ontario	43 (7.1)	2194 (7.1)
r/Winnipeg	34 (5.6)	1379 (4.4)
r/onguardforthee	34 (5.6)	960 (3.1)
r/ottawa	31 (5.1)	1701 (5.5)
r/alberta	25 (4.1)	1949 (6.3)
r/VictoriaBC	25 (4.1)	890 (2.9)
r/NovaScotia	23 (3.8)	648 (2.1)
r/Edmonton	23 (3.8)	656 (2.1)
r/CanadaPolitics	21 (3.5)	545 (1.8)
r/Canada_sub	21 (3.5)	1671 (5.4)
r/Calgary	20 (3.3)	933 (3.0)
r/newfoundland	17 (2.8)	334 (1.1)
r/toronto	14 (2.3)	1575 (5.1)
r/saskatchewan	12 (2.0)	880 (2.8)
r/saskatoon	12 (2.0)	532 (1.7)
r/PEI	12 (2.0)	213 (0.7)
r/montreal	7 (1.2)	371 (1.2)
r/StJohnsNL	6 (1.0)	67 (0.2)
r/Manitoba	6 (1.0)	142 (0.5)
r/newbrunswickcanada	4 (0.7)	145 (0.5)
r/moncton	3 (0.5)	23 (0.1)
r/regina	2 (0.3)	75 (0.2)
r/fredericton	2 (0.3)	9 (<0.01%)
r/Yukon	1 (0.2)	33 (0.1)
r/NWT	1 (0.2)	9 (<0.01%)
r/Whitehorse	1 (0.2)	0 (0.0)

a Subreddit names are presented as originally created on Reddit. Capitalization varies across communities and does not necessarily reflect conventional geographic naming standards.

### Topic Analysis

We extracted 14 meaningful topics from 1111 of the documents, characterized by their respective scores (Table 4). However, 241 documents were associated with a mixture of topics or contained topics that were not clearly recognizable and, as such, were invalid topics (topic -1 in Table 4). The overall topic distribution across all 1352 documents is presented in Table 4. Heating and cooling mechanics and costs associated with each appeared frequently throughout the topics.

**Table 4.** Topic and top word distribution across documents (n=1111 valid topics).

Topic	Top words	Values, n (%)
-1 (invalid topic)	—^a^	241 (17.8)^b^
0	pump, furnace, gas, heat, house	571 (51.4)^c^
1	weather, climate, rain, records, wave	218 (19.6)^c^
2	[air] conditioners, people, health...	51 (4.6)^c^
3	tax, carbon, moe, saskatchewan, money	42 (3.7)^c^
4	walmart, prices, food, grocery, costco	40 (3.6)^c^
5	landlord, rent, tenants, building...	35 (3.2)^c^
6	ac, windows, curtain, fans, sleep	29 (2.6)^c^
7	cat, dog, pets, fan, ice	26 (2.3)^c^
8	wear, hoodie, pants, wool, shoes	23 (2.1)^c^
9	grid, power, nuclear, shortages...	21 (1.9)^c^
10	valve, condo, baseboard, apartment...	19 (1.7)^c^
11	cool, pillow, ice, pools, healthlink	15 (1.4)^c^
12	love, hate, like, humidity, outside	11 (1.0)^c^
13	schools, manitoba, strike, grade...	10 (0.9)^c^

a Invalid topic excluded from the top word analysis.

b Denominator is all documents, n=1352.

c Denominator is all valid documents, n=1111.

### Sentiment Analysis

**Figure 1.**
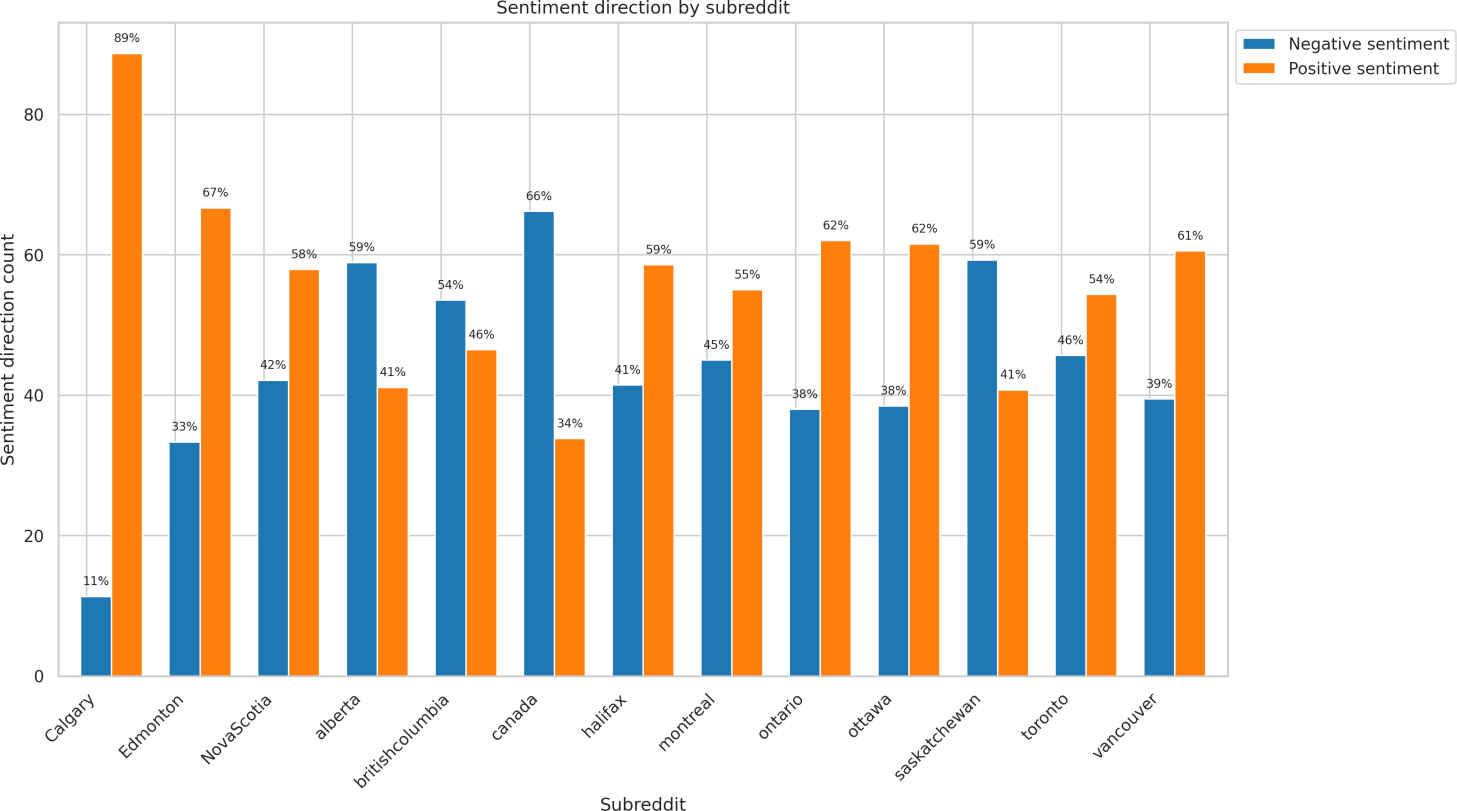
Distribution of negative and positive sentiment documents across top 13 subreddits.

Figure 1 illustrates the distribution of negative and positive sentiment documents across the top 13 subreddits (the largest subreddits with a comparable number of documents). The subreddit r/calgary (positive: n=47, 89% vs negative: n=6, 11%) had the highest positive sentiment, followed by r/Edmonton (n=32, 67% vs n=16, 33%) and r/ontario (n=80, 62% vs n=49, 38%). The subreddits with the highest negative sentiment included r/canada (positive: n=47, 34% vs negative: n=92, 66%), followed by r/saskatchewan (n=11, 41% vs n=16, 59%) and r/alberta (n=30, 41% vs n=43, 59%).

### Emotion Analysis

**Figure 2.**
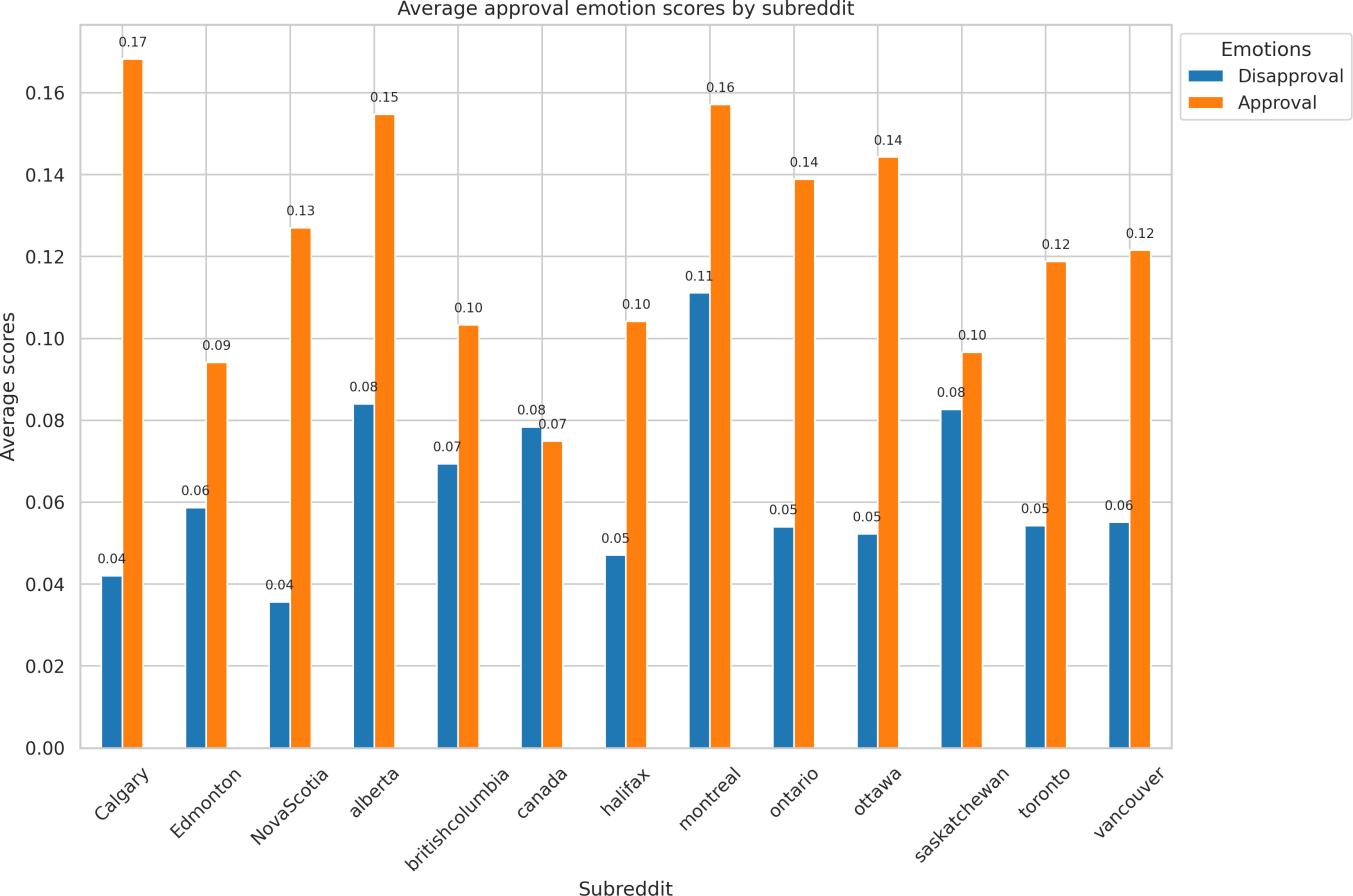
Approval and disapproval emotion distribution by top 13 subreddits.

We discerned diverse emotions within the top 13 subreddits (the largest subreddits with a comparable number of documents). Figure 2 displays the average scores for the emotions of approval and disapproval across each subreddit. Within the subreddits, r/Calgary (0.17) had the highest approval emotion score, followed by r/montreal (0.16) and r/alberta (0.15). However, r/montreal (0.11) had the highest disapproval emotion score, followed by r/alberta (0.084), and r/saskatchewan (0.083).

**Figure 3.**
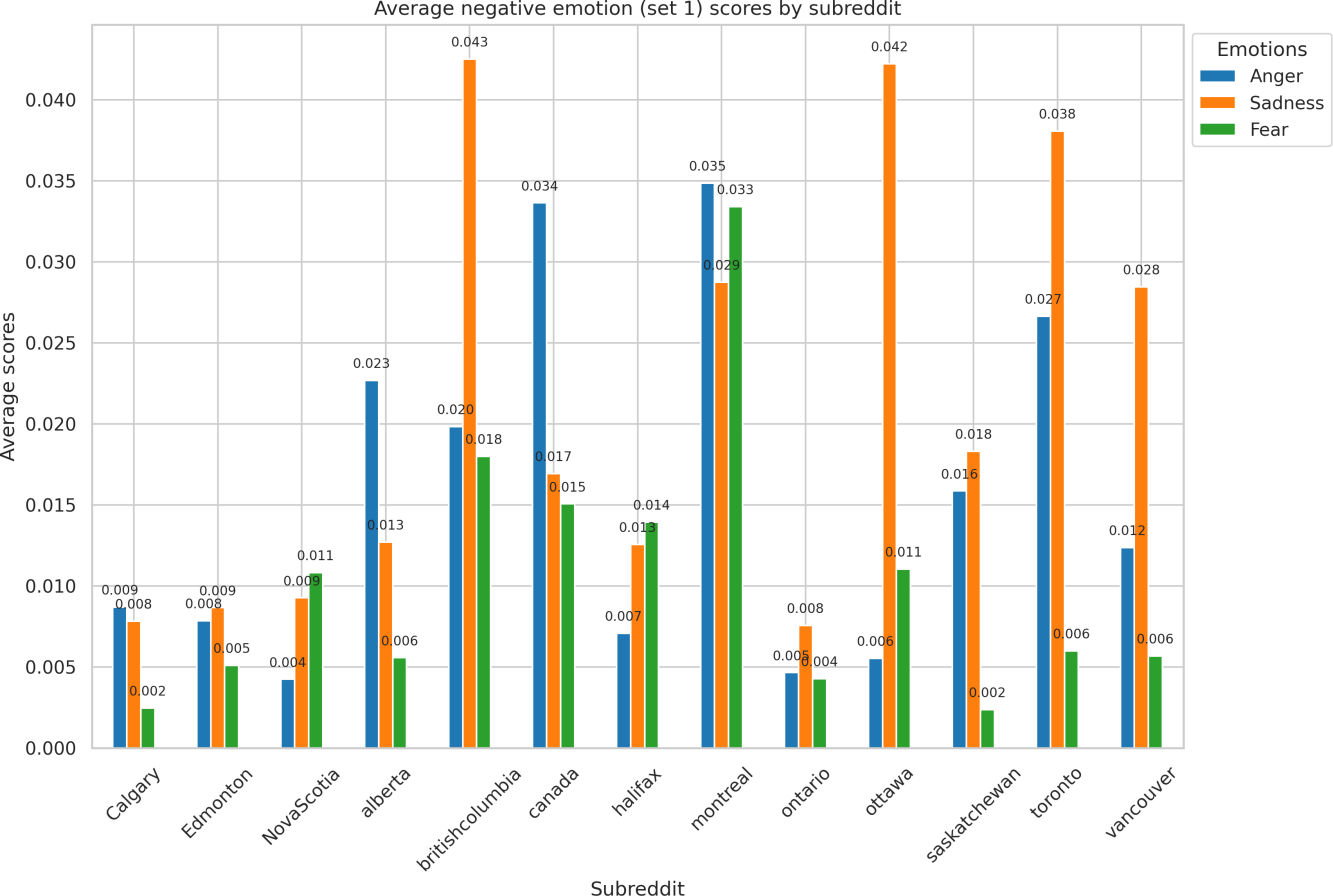
Negative emotion (anger, sadness, and fear) distribution by top 13 subreddits.

Similarly, Figure 3 presents the average scores for anger, sadness, and fear across the top 13 subreddits. Anger was the highest in r/montreal (0.035), r/canada (0.034), and r/toronto (0.027). Sadness was the highest in r/britishcolumbia (0.043), r/ottawa (0.042), and r/toronto (0.038). Finally, fear was the highest in r/montreal (0.033), r/britishcolumbia (0.018), and r/canada (0.015).

**Figure 4.**
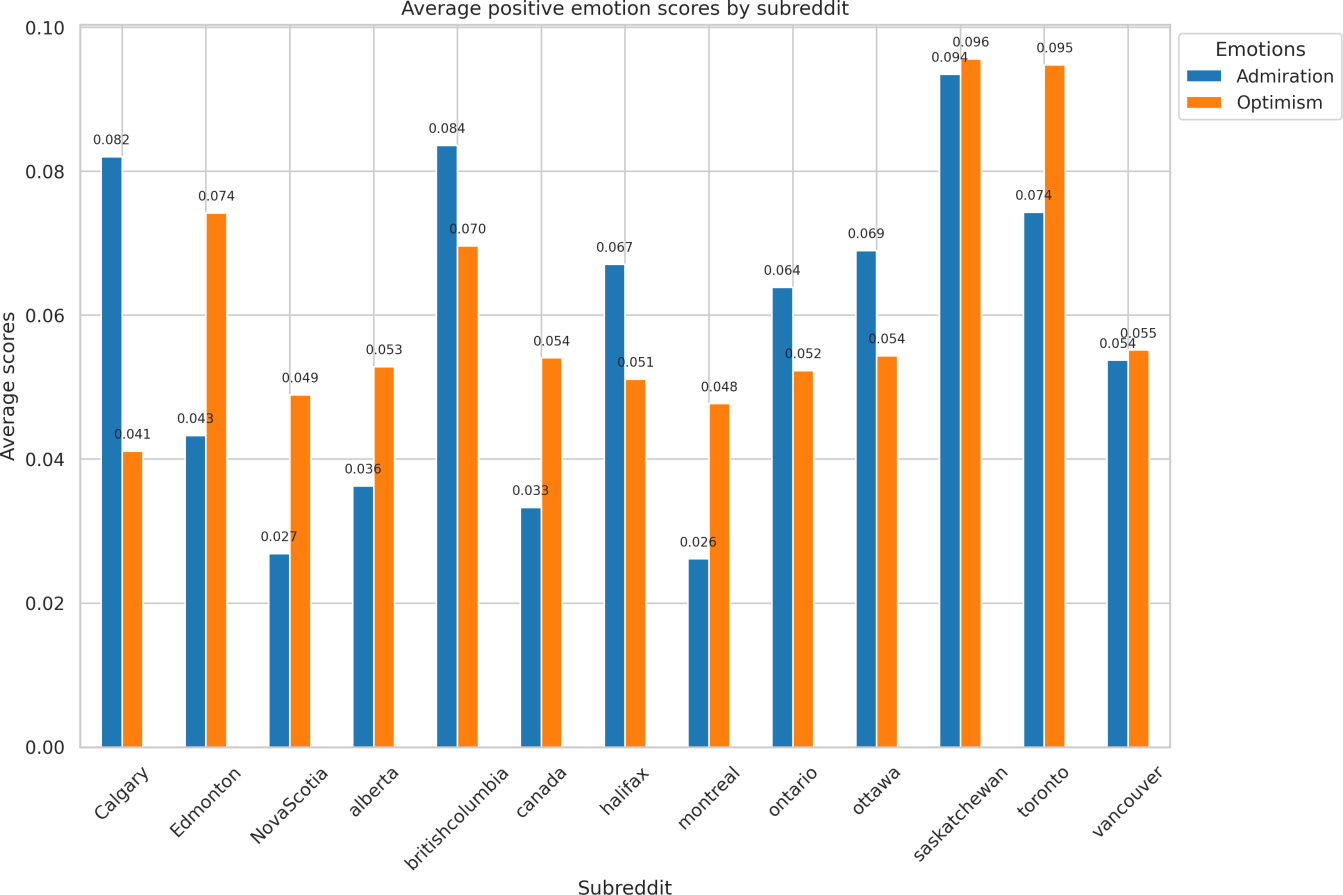
Positive emotion (admiration and optimism) distribution by top 13 subreddits.

Figure 4 illustrates the average scores for admiration and optimism for the top 13 subreddits. Admiration was found to be the highest in r/saskatchewan (0.094), r/britishcolumbia (0.084), and r/Calgary (0.082). Optimism was the highest in r/saskatchewan (0.096), r/toronto (0.095), and r/Edmonton (0.074).

### Engagement Analysis

Table 5 illustrates the average upvote score for the posts and comments extracted from the top 13 subreddits, reflecting the levels of community engagement and approval. In our dataset, the subreddit r/toronto (23.68) had the highest level of engagement and approval, followed by r/canada (17.87) and r/montreal (17.14).

**Table 5.** Average normalized upvote by top 13 subreddits.

Subreddit	Upvote number
r/toronto	23.686033
r/canada	17.872152
r/montreal	17.13952
r/britishcolumbia	15.612242
r/vancouver	14.76366
r/ottawa	12.568449
r/Calgary	12.30941
r/alberta	11.324873
r/saskatchewan	9.57762
r/ontario	9.345084
r/Edmonton	6.49853
r/Halifax	6.349839
r/NovaScotia	5.056377

## Discussion

### Principal Findings

This study examined Reddit discussions related to extreme heat in Canada to identify common topics and concerns, explore sentiment and emotional responses, and analyze how these relate to community engagement. The findings indicate that extreme heat and climate change are prominent themes among Canadian Reddit users, with substantial regional variation in both the discussion focus and emotional tone. Engagement patterns coincided with discussions of climate-related issues, suggesting that social media can provide signals of public perception and discourse about extreme weather events. Interpreted through a risk communication lens, these discussions reflect how people perceive health risks, express uncertainty, and negotiate adaptation strategies in an online environment. From an information ecosystem perspective, Reddit acts as a dynamic venue where public health–relevant information is coconstructed, contested, and amplified. While our findings suggest that Reddit can surface emerging concerns and perspectives related to extreme heat, these insights should be understood as complementary to other forms of evidence (eg, surveys, interviews, or other social media analyses) rather than representative of public perceptions in Canada as a whole.

Reddit can provide an exploratory view of public perceptions, particularly among users who are active on the platform, especially with regard to current events that impact health. Prior research has highlighted its utility for tracking perceptions during events such as influenza outbreaks [30]. In this study, Reddit discussions captured public concerns about extreme heat and its growing threats to health, safety, and economic well-being, particularly in cities such as Halifax and Vancouver. Users referenced real-world impacts such as elevated mortality following heatwaves, heightened fire risks, and deteriorating quality of life, which align with national data on climate-related health outcomes [7]. These discussions may reflect broader concerns about climate anxiety among some Canadians, with many linking local events to larger concerns about global climate change [31]. The themes we identified, such as concerns about energy costs, adaptation strategies, and climate anxiety, should be viewed as potential information needs. They may help guide future research and message development, but actionable public health communication strategies will require additional data sources and direct engagement with communities.

### Common Discussion Topics and Concerns About Extreme Heat in Canada

Reddit users across Canada expressed widespread interest in heat-related issues, as reflected in the dominance of heat-related content in the analyzed posts. Specifically, 82.5% (n=501) of the posts and over 80% (n=25,253) of the comments centered on the topic of “heat.” In addition to general heat concerns, other common themes included extreme heat, hot weather, and hot summer. These patterns align with national polling data, such as a recent Leger survey showing that 70% of Canadians are concerned about climate change and its role in increasing extreme weather events [31].

The r/Canada subreddit had the highest number of posts, comments, and engagement overall, indicating the breadth of public discourse about extreme heat and climate change across the country. Region-specific subreddits, such as r/halifax and r/britishcolumbia, followed closely, with high volumes of posts and engagement—likely reflecting local experiences with recent extreme heat events in regions historically known for more moderate climates [32]. These observations parallel other Reddit-based studies on climate discourse that similarly found widespread concern about the impacts of climate change, with the consequences of climate change a main topic of discussion on 100,000 Reddit posts [33].

Beyond general climate concerns, the topic modeling analysis revealed several dominant themes related to daily life and broader societal challenges. The largest topic cluster focused on heating and cooling, including practical terms, such as “pump,” “furnace,” and “gas.” These reflect public concern over energy infrastructure and affordability, particularly in the context of extreme temperatures. This is supported by evidence showing that many Canadian households face energy poverty, leading to discomfort, health risks, and financial strain [34]. Reddit users expressed anxiety around increasing energy costs and insufficient cooling infrastructure, especially during heatwaves.

Health impacts emerged as another important discussion topic. Climate change and extreme heat are contributing to health inequities across the determinants of health, leading to impacts on community and individual health in Canada [35]. A popular news article in 2024 focused on extreme heat and outlined how racialized communities, those renting their homes, and those living in energy poverty were found to be at higher risk [36]. These findings correspond with the broader recognition of how climate change intersects with social determinants of health [35] and demonstrate how Reddit discussions mirror evolving media, political, and institutional attention to these equity concerns.

### Attitudes and Emotions Expressed in Reddit Discussions on Extreme Heat

The emotional tone of Reddit discussions varied substantially by region, illustrating different levels of concern, belief, and lived experience with climate change across the country. Subreddits such as r/Calgary and r/Edmonton showed more positive sentiment overall, while r/canada and r/saskatchewan leaned toward more negative sentiment. This contrast highlights the nuanced ways that attitudes toward climate-related challenges manifest online.

Regional differences in emotion were also apparent in the emotion analysis. For example, subreddits such as r/saskatchewan and r/calgary expressed relatively high levels of admiration and optimism. This may reflect regional differences in belief systems about climate change. Some research suggests that individuals who downplay the severity of climate change tend to report more positive feelings, which can lead to a phenomenon known as “denial-based hope” [37]. While this outlook may reduce perceived urgency, it can also hinder individual and collective action to address climate risks [38].

In contrast, negative emotions, such as anger, sadness, and fear, were more pronounced in subreddits such as r/montreal, r/britishcolumbia, and r/canada.

These emotions may stem from direct experiences with climate-related disasters, such as the 2021 heat dome in British Columbia and the 2023 wildfires in Nova Scotia. Consistent with previous research, negative emotional responses, particularly fear, were associated with heightened climate awareness and a greater likelihood of supporting or engaging in adaptive action [39]. In our dataset, these emotions appeared in ways that may align with those patterns, though our study did not measure behavior directly. Sustained exposure to such distress can also lead to climate-related mental health challenges. A recent survey found that 78% of young Canadians reported negative mental health impacts from climate change, underscoring the psychological burden of climate anxiety [40].

### Engagement Patterns in Heat-Related Reddit Discussions: Connections With Topics and Sentiment

Community engagement, as measured by upvotes, was the highest in subreddits r/toronto and r/canada, indicating high levels of user participation and approval in discussions surrounding extreme heat. Upvotes make a post more visible on Reddit and show others’ support or approval of the topic [29].

These findings reflect broader trends in climate change communication on social media, where approximately one-third of users engage with climate-related content, particularly Gen Z adults [41]. Engagement is often higher for posts that include calls to action or connect the problem of climate change with potential solutions [41]. Credible sources, linking the problem to solutions, and including calls to action are key best practices for engaging people in climate change information [42]. In this context, high engagement with Reddit content may indicate a public appetite for action-oriented or relatable discussions, especially when aligned with credible sources and practical implications for everyday life (eg, energy costs, health impacts).

Engagement levels also intersected with topic and sentiment trends. Subreddits with high discussion volumes and strong negative emotional expression also saw high levels of engagement. This pattern indicates that emotionally charged posts may coincide with more active participation, though causality cannot be inferred. While our results suggest that stronger emotional expression often coincided with higher engagement, our study was not designed to explain why some emotionally charged posts gain more traction than others. Understanding these dynamics would require qualitative analysis of discourse and context, or triangulation with other data sources.

Taken together, these findings suggest that Reddit can serve as an exploratory lens into how Canadians discuss and react to extreme heat, surfacing concerns that range from household-level adaptation strategies to broader issues of affordability, energy systems, and equity. While not representative of the population as a whole, these insights highlight potential information needs and public concerns that public health agencies and policymakers may wish to consider alongside other information sources when designing communication strategies around extreme heat and climate risks. More broadly, this study illustrates how computational analyses of social media can complement traditional research approaches by identifying emergent themes and emotions in real time, thereby offering early signals of public discourse that warrant further investigation. This research demonstrates the value of integrating social media monitoring into the broader toolkit for public health communication and climate resilience planning.

### Future Research

Future research should examine Reddit posts related to extreme heat and climate change in Canada for misinformation or disinformation. Within this research, it would be beneficial to examine how misinformation or disinformation about climate change and extreme heat impacts the engagement and emotions found within posts. Further exploring the relationship between emotions and action in climate adaptation would also help shape initiatives. Developing communication reflective of the topics and discussions found in the subreddits and evaluating them across communities would also help understand the utility of social media discourse for informing official messaging. Future research should also build on this descriptive foundation by incorporating qualitative coding of excerpts, deeper contextual interpretation, and comparisons with external datasets, such as meteorological records or health outcomes. Finally, discourse found on Reddit should be compared with other social media platforms and Google trends to understand similarities and differences found in online sources.

### Limitations

This research was based on the social media platform Reddit and common subreddits in Canada. Generalizability is limited because Reddit users skew toward younger demographics and each subreddit has its own norms, moderation practices, and community perspectives. As Caplan and Purser [43] highlight, subreddit-specific cultures and sociotechnical contexts shape discourse; our inclusion of 30 national, provincial or territorial, and local subreddits was intended to mitigate this, but the findings should still be interpreted as a broad snapshot rather than as representative of each subreddit’s culture. Future research could extend this work by analyzing subreddit-specific practices in greater depth and comparing results with other online platforms.

A key technical limitation is Reddit’s API restriction, which caps retrieval at 1000 posts per query. In high-traffic subreddits, this constraint can bias the dataset toward more recent content and limit temporal coverage.

Model-related limitations must also be considered. The BERTopic and sentiment or emotion models used were trained on general-purpose corpora that differ from Canadian Reddit discussions of extreme heat. This may lead to systematic misclassification; for example, sarcasm, humor, or colloquial expressions may be interpreted incorrectly. These constraints affect both topic coherence and sentiment assignments, underscoring the need for caution in drawing substantive conclusions.

Finally, despite filtering out bot-generated, deleted, and non-English comments, undetected bots remain a persistent challenge. Sophisticated bots can mimic human discourse and potentially amplify particular narratives, subtly influencing topic distributions and sentiment profiles. This is especially relevant in politically and socially salient discussions.

### Conclusions

This study demonstrates the impacts of extreme heat and climate-related challenges across Canadian communities, found within subreddit discussions. Topics around heating and cooling, financial burdens, and broader social implications of adapting to a changing climate were found. Community engagement was particularly high in r/toronto and r/Canada, signifying active discourse and collective concern. The emotional analysis showed regional differences, with positive emotions such as optimism and admiration common in r/saskatchewan and r/Calgary, and negative emotions such as fear and anger more common in r/montreal and r/canada. Terms such as “pump,” “costs,” and “climate” were common and reinforced the need for adaptive strategies that address social, economic, and environmental concerns. Overall, the findings demonstrate how Reddit may serve as a useful exploratory platform for examining discourse around climate change and extreme heat, offering signals that could inform future tailored and community-specific communication efforts.

## Abbreviations

API: application programming interface
BERTopic: bidirectional encoder representations topic modeling
